# Comparing sensitivity to change of two patient-reported outcome measures in a randomised trial of patients referred for physiotherapy services

**DOI:** 10.1186/1745-6215-14-S1-O50

**Published:** 2013-11-29

**Authors:** Selman Mirza, Chris Salisbury, Cherida Hopper, Nadine Foster, Alan Montgomery

**Affiliations:** 1 University of Bristol, Bristol, UK; 2 Keele University, Keele, UK; 3 University of Nottingham, Nottingham, UK

## 

The SF-36 Physical Component Summary (PCS) and the Measure Yourself Medical Outcome Profile (MYMOP) are respectively generic and individualised self-report tools to assess health. The aim of this study was to compare responsiveness to change in these outcomes among patients with musculoskeletal problems referred for physiotherapy.

Data were collected at baseline, 6 weeks and 6 months from 3714 participants recruited to the PhysioDirect randomised trial. Utilising change from baseline scores among participants responding as ‘slightly better’ on a global item, three different response statistics – standardised response mean, effect size, Guyatt’s response index – were calculated for MYMOP and PCS. These were formally compared using a modified jacknife procedure.

MYMOP response statistics were 0.88, 1.08 and 1.11 at 6 weeks and 0.83, 1.16 and 1.16 at 6 months for SRM, ES and GRI respectively. Values for PCS were 0.57, 0.50 and 0.70 at 6 weeks and 0.63, 0.62 and 0.86 at 6 months. There was strong evidence that MYMOP was more sensitive to change than PCS at both time points (p<0.001 for all comparisons). There was evidence that MYMOP was more responsive among women than men at 6 weeks but not at 6 months, and that both MYMOP and PCS were more sensitive among younger than older participants at both times points.

MYMOP was more responsive to change than PCS, and responsiveness remained more stable over time. Allowing trial participants to define and measure symptoms that cause them greatest problems may allow detection of smaller but clinically important effects than generic outcomes.

